# Prometheus’s Fire, Part III: The Forge of Hephaestus—Non-Pharmacological and Pharmacological Interventions Targeting Adipose Tissue Immunometabolism in Obesity

**DOI:** 10.3390/ijms27219692

**Published:** 2026-10-29

**Authors:** Anna Grudniewska, Beata Mrozikiewicz-Rakowska, Kacper Wiszniewski, Krzysztof J. Filipiak, Alina Kuryłowicz

**Affiliations:** 1 Lux Med Medical Centre in Warsaw, 49 Komitetu Obrony Robotników Street, 02-146 Warsaw, Poland; grudniewska.anna@gmail.com; 2 Department of Endocrinology, Centre of Postgraduate Medical Education, Marymoncka Street 99/103, 01-813 Warsaw, Poland; kacpor94@gmail.com; 3 Centre of Postgraduate Medical Education, Marymoncka Street 99/103, 01-813 Warsaw, Poland; krzysztof.filipiak@cmkp.edu.pl; 4 Department of General Medicine and Geriatric Cardiology, Medical Centre of Postgraduate Education, ul. Czerniakowska 231, 00-416 Warsaw, Poland; alina.kurylowicz@cmkp.edu.pl

**Keywords:** adipose tissue immunometabolism, NLRP3 inflammasome, GLP-1 receptor agonists, tirzepatide, caloric restriction, Mediterranean diet, bariatric surgery, SGLT2 inhibitors, metformin, obesity

## Abstract

There is a growing recognition of obesity as a chronic immunometabolic disease in which impaired adipose tissue expandability contributes to a self-amplifying inflammatory network that extends across multiple organs. This state-of-the-art narrative review, the third and final part of the Prometheus’s Fire series, considers how lifestyle interventions, bariatric surgery and pharmacological therapies may disrupt this network and support the restoration of immunometabolic homeostasis. We introduce the concept of therapeutic convergence, describing how interventions with different primary mechanisms can influence shared processes, including NOD-, LRR- and pyrin domain-containing protein 3 (NLRP3) inflammasome activity, adipokine dysregulation, adipose tissue remodelling, mitochondrial stress and inter-organ inflammatory signalling. Evidence from randomised controlled trials, meta-analyses and mechanistic studies shows that these interventions differ considerably in the extent and breadth of their effects. Bariatric surgery, glucagon-like peptide-1 (GLP-1) receptor agonists and double GLP-1/glucose-dependent insulinotropic polypeptide (GIP) receptor agonists (tirzepatide) have the most consistent evidence for coordinated effects on adipose tissue inflammation, metabolic dysfunction and inter-organ signalling. Other interventions appear to influence more selected components of the network and may therefore have greater value as complementary rather than comprehensive approaches. This perspective also highlights a limitation of relying on body weight as the primary measure of therapeutic success. Weight loss remains an important clinical goal, but it may not fully reflect the extent or durability of immunometabolic recovery. The therapeutic convergence framework therefore offers a way to understand how mechanistically distinct interventions can produce overlapping systemic benefits and may help identify treatment strategies that more effectively restore adipose tissue function while disrupting the inflammatory processes that sustain metabolic disease.

## 1. Introduction

Obesity is increasingly recognised as a chronic immunometabolic disease rather than a disorder defined solely by excessive adipose tissue accumulation. A central pathological event is the progressive loss of adipose tissue expandability, which promotes adipocyte stress, immune-cell recruitment, mitochondrial and endoplasmic reticulum dysfunction, inflammasome activation and disruption of adipokine signalling. These processes are not confined to adipose tissue. Through endocrine, metabolic and inflammatory communication, they can propagate to the liver, skeletal muscle, pancreatic β-cells, perivascular adipose tissue and other vulnerable organs, creating a self-amplifying network of metabolic and inflammatory dysfunction [1,2,3].

The previous parts of the Prometheus’s Fire series established the mechanistic basis of this concept. Part I described how sustained nutrient excess and impaired adipose tissue expandability promote mitochondrial dysfunction, cellular stress, NOD-, LRR- and pyrin domain-containing protein 3 (NLRP3) inflammasome activation and adipokine dysregulation. Part II extended this framework beyond adipose tissue, showing how local inflammatory disturbances can propagate through organ-specific and inter-organ feed-forward loops involving the liver, skeletal muscle, pancreatic β-cells, perivascular adipose tissue and the tumour microenvironment. Together, these processes form the self-amplifying multi-organ inflammatory network termed the Hydra of Inflammation.

The present, third part examines whether, and through which mechanisms, the self-amplifying immunometabolic network described in Parts I and II can be therapeutically attenuated or interrupted.

Lifestyle interventions, bariatric surgery and pharmacological therapies differ substantially in their primary mechanisms. Caloric restriction alters nutrient availability; exercise modifies energy flux, mitochondrial function and skeletal muscle–adipose communication; bariatric surgery produces profound and sustained metabolic remodelling; and pharmacological agents act through diverse hormonal, metabolic and inflammatory pathways. Nevertheless, accumulating evidence indicates that their downstream effects converge on a partially overlapping set of biological processes. These include attenuation of NLRP3 inflammasome activity and inflammatory signalling, improvement of adipokine balance, reduction of adipocyte stress and maladaptive tissue remodelling, restoration of mitochondrial and metabolic function, and weakening of pathological communication between adipose tissue and other organs.

This therapeutic convergence is particularly relevant to obesity because no single molecular pathway fully accounts for the complexity of the disease. For example, reduction of adipose tissue mass may decrease inflammatory signalling, but the biological consequences of weight loss also depend on changes in adipocyte size, tissue remodelling, immune-cell composition, adipokine secretion and metabolic substrate handling. Conversely, interventions with relatively modest effects on body weight may nevertheless modify selected components of the inflammatory network. The clinical phenotype observed after treatment therefore reflects the combined consequences of multiple interconnected processes rather than the modulation of a single pathway.

Accordingly, therapeutic interventions should not be viewed solely according to their primary pharmacological target or their capacity to reduce body weight. A systems-level perspective allows their effects to be considered in relation to the network established in Parts I and II: the adipose tissue inflammatory state, the balance of adipose-derived signals, tissue expandability and remodelling, cellular metabolic stress, and the inter-organ feedback loops that sustain chronic inflammation. Within this framework, the previously identified four biological nodes, NLRP3 inflammasome activation, adipokine homeostasis, adipose tissue expandability and inter-organ inflammatory coupling, remain useful as conceptual reference points, but they should not be interpreted as discrete or mutually exclusive therapeutic targets. Rather, they represent interconnected components of a broader immunometabolic system.

The extent to which individual interventions influence these components is heterogeneous. Some therapies act predominantly on inflammatory or metabolic stress pathways, whereas others produce broader changes encompassing adipose tissue remodelling, endocrine signalling and inter-organ communication. Of note, evidence for these effects varies in strength and derives from different experimental and clinical settings. Therefore, the present review does not propose a rigid ranking of therapies according to the number of biological nodes they engage. Instead, it examines the breadth, direction and level of evidence for network modulation produced by each intervention.

The aim of this review is to integrate current evidence on lifestyle interventions, bariatric surgery and pharmacological therapies within the immunometabolic network model developed in Parts I and II of the Prometheus’s Fire series. For each intervention, we consider its primary mechanism together with downstream effects on adipose tissue biology, inflammatory signalling, adipokine function, cellular stress and inter-organ communication. Particular attention is given to whether therapeutic effects extend beyond weight reduction to modify the biological processes that sustain chronic inflammation.

From this perspective, effective obesity treatment may be understood not simply as the reduction of excess adiposity but as the progressive restoration of adipose tissue immunometabolic quality and attenuation of the self-amplifying signals that maintain systemic metabolic inflammation. Therapeutic convergence therefore provides a conceptual bridge between apparently distinct interventions and the shared biological improvements observed across obesity treatment strategies.

### Literature Search and Review Approach

A literature search was conducted in PubMed/MEDLINE and Scopus for publications from January 2019 to August 2026, using combinations of the following search terms: caloric restriction NLRP3, Mediterranean diet inflammation, PREDIMED, CORDIOPREV, exercise adipokines, exercise macrophage polarisation, bariatric surgery adipose tissue inflammation, sleep deprivation NLRP3, circadian rhythm adipokines, metformin AMPK inflammation, SGLT2 inhibitors NLRP3, GLP-1 receptor agonist adipose tissue macrophage, tirzepatide adipose tissue, tirzepatide GIP receptor, semaglutide MASH, SYNERGY-NASH, ESSENCE trial, SUMMIT trial, SURPASS-CVOT, STEP-HFpEF, SELECT trial, PPARγ agonist adipose expandability, statins anti-inflammatory, omega-3 inflammation, DPP-4 inhibitors NLRP3, and immunometabolic convergence obesity. Priority was given to randomised controlled trials, cardiovascular and hepatic outcome trials, mechanistic studies in human adipose tissue, and high-quality systematic reviews and meta-analyses addressing the immunometabolic effects of pharmacological and lifestyle interventions in obesity. The literature search was designed to prioritise contemporary evidence from 2019 to 2026, while selected earlier studies were retained where they provided foundational clinical or mechanistic evidence. Pre-2019 references were retained selectively in three categories: foundational cardiovascular outcome trials establishing the cardiovascular safety and/or clinical effects of the respective drug classes, including LEADER, SUSTAIN-6, and SAVOR-TIMI 53; mechanistic studies providing foundational evidence for the anti-inflammatory effects of statins, omega-3 fatty acids, and DPP-4 inhibitors; and the primary experimental study characterising GLP-1-mediated suppression of adipose tissue macrophage infiltration, which remains directly relevant to the mechanism discussed herein. As this article is designed as a state-of-the-art narrative review, the literature was synthesised thematically rather than according to a systematic-review protocol. The selection and interpretation of evidence were guided by mechanistic relevance, clinical significance, methodological quality, and thematic coherence with the therapeutic convergence framework developed herein. Preferred Reporting Items for Systematic Reviews and Meta-Analyses (PRISMA) methodology was not applied because the present work is a narrative, state-of-the-art synthesis rather than a systematic review or meta-analysis.

## 2. Non-Pharmacological Interventions

Lifestyle interventions remain the foundation of obesity treatment, not simply because they reduce body weight, but because they directly target the biological mechanisms described in Parts I and II of this series. Despite their apparent diversity, caloric restriction, the Mediterranean diet, physical activity and bariatric surgery share a common molecular signature: suppression of NLRP3 inflammasome activity, restoration of adiponectin-mediated homeostatic control, promotion of M1 → M2 macrophage reprogramming and improvement of mitochondrial function [1,2,3,4,5,6,7,8].

### 2.1. Dietary Interventions

#### 2.1.1. Caloric Restriction

Caloric restriction targets the fundamental pathophysiological process underlying obesity-associated metabolic complications: the chronic low-grade inflammation of dysfunctional adipose tissue [1,2,3]. Its immunometabolic effects are mediated through molecular mechanisms that extend beyond the consequences of weight loss alone.

Anti-inflammatory effects of caloric restriction have been demonstrated across different populations. Notably, the CALERIE Phase 2 trial demonstrated meaningful reductions in systemic inflammatory markers even in non-obese adults, suggesting that the immunometabolic benefits of caloric restriction are not contingent on a baseline diagnosis of obesity [9].

Caloric restriction (CR) constitutes the primary dietary intervention in obesity, exerting well-documented immunometabolic effects through complementary molecular mechanisms. In preclinical models of diet-induced obesity, short-term moderate caloric restriction activates the AMP-activated protein kinase (AMPK) → sirtuin 1 (SIRT1) → PPARγ coactivator 1α (PGC-1α) signalling axis, reduces reactive oxygen species (ROS) generation, and suppresses nuclear factor κB (NF-κB) protein levels in white adipose tissue and liver, effects observed after as little as four weeks of 25% energy restriction [10].

The most direct clinical evidence comes from the CALERIE Phase 2 trial, a randomised controlled trial of 25% caloric restriction maintained for 24 months in 218 non-obese adults. Despite achieving an actual mean restriction of approximately 12% over the full intervention period, participants exhibited a significant reduction in circulating C-reactive protein (CRP) (approximately 40%) and tumour necrosis factor α (TNF-α) (approximately 50%) compared with ad libitum controls, effects that were not fully explained by the degree of weight loss achieved [9]. These findings support the view that CR engages anti-inflammatory pathways through mechanisms that extend beyond simple reduction in fat mass, consistent with the AMPK → SIRT1 → PGC-1α → NF-κB signalling cascade demonstrated in preclinical models [10]. Notably, participants in the CALERIE trial also demonstrated improvements in cardiovascular risk factors, including reductions in blood pressure and favourable changes in lipid profile, suggesting that CR-induced suppression of chronic low-grade inflammation may contribute to a reduction in obesity-related cardiovascular risk [9].

It should be noted, however, that caloric restriction is contraindicated in patients with a history of disordered eating, and its implementation requires careful clinical assessment; the psychological and behavioural risks of sustained energy restriction fall outside the mechanistic scope of the present review but are important considerations in clinical practice.

The anti-inflammatory effects of caloric restriction vary depending on the degree of energy deficit and the duration of the intervention. Preclinical data indicate that significant suppression of NF-κB-dependent inflammation is achievable within weeks of moderate restriction [10], whilst the CALERIE Phase 2 trial demonstrates that sustained 24-month restriction produces clinically meaningful reductions in CRP and TNF-α in humans [9].

#### 2.1.2. Mediterranean Diet

What distinguishes the Mediterranean diet from other dietary patterns is not primarily its macronutrient composition but its capacity to recalibrate the adipokine secretory axis without requiring substantial caloric restriction. Polyphenols such as resveratrol, quercetin and hydroxytyrosol activate SIRT1 and AMPK in adipocytes, driving adiponectin gene expression and simultaneously reducing NF-κB-dependent leptin secretion. The practical implication is that Mediterranean diet adherence can partially restore adipose tissue endocrine function, addressing what Parts I and II identified as the loss of the adiponectin-mediated homeostatic brake on NLRP3 activity, even in patients who do not achieve significant weight loss [11,12].

Beyond its nutritional composition, the Mediterranean diet functions as an immunometabolic intervention capable of attenuating chronic inflammatory signalling through multiple molecular pathways. Rich in polyphenols, monounsaturated fatty acids, omega-3 fatty acids and dietary fibre, it suppresses NF-κB activation, reduces circulating IL-6 and TNF-α, increases adiponectin secretion and promotes M2 macrophage polarisation [5,11,12].

The Mediterranean diet (MedDiet) exerts documented anti-inflammatory effects extending beyond simple caloric restriction, operating through several specific molecular mechanisms. Polyphenols and oleocanthal present in extra-virgin olive oil act as natural inhibitors of Toll-like receptor 4 (TLR4)/NF-κB signalling in adipocytes, reducing transcriptional priming of NLRP3 and the production of pro-inflammatory cytokines [5,11,12]. The high content of soluble and fermentable fibre promotes the growth of short-chain fatty acid (SCFA)-producing microbiota; the resulting increase in luminal and systemic short-chain fatty acid (SCFA) concentrations activates G protein-coupled receptor 43 (GPR43) receptors on adipocytes and macrophages, thereby attenuating NF-κB-dependent NLRP3 priming and promoting M1 → M2 macrophage polarisation [5]. Omega-3 fatty acids abundant in the Mediterranean diet activate the G protein-coupled receptor 120 (GPR120) receptor and stimulate the biosynthesis of maresins and resolvins—lipid mediators that directly inhibit caspase-1 and reduce the production of interleukin-1β (IL-1β) and IL-18 [5,11]. Long-term data from the PREDIMED trial demonstrated sustained reductions in inflammatory markers, including high-sensitivity CRP (hs-CRP), interleukin-6 (IL-6) and TNF-α, during Mediterranean diet intervention [11]. A subsequent analysis from the CORDIOPREV study demonstrated that the Mediterranean diet is superior to a low-fat diet in reducing visceral adipose tissue and improving the systemic adipokine profile [12].

The PREDIMED trial enrolled 7447 adults at high cardiovascular risk [11], while the CORDIOPREV study included 1002 patients with established coronary heart disease [12]; together, these randomised controlled trials provide the principal clinical evidence base for the anti-inflammatory effects of the Mediterranean diet in high-risk populations.

### 2.2. Physical Activity

#### Aerobic and Resistance Exercise

Exercise as an Immunometabolic Modulator. The classical description of exercise as a treatment for insulin resistance underestimates its biological scope. Regular aerobic and resistance training activates AMPK across multiple tissues simultaneously in adipocytes suppressing thioredoxin-interacting protein (TXNIP)-NLRP3 coupling, in skeletal muscle improving mitochondrial quality control, and in macrophages promoting M2 polarisation through PGC-1α-dependent pathways. The exercise-induced release of IL-6 from contracting myocytes is a case in point: although IL-6 is typically regarded as a pro-inflammatory cytokine in the context of adipose tissue dysfunction, the transient IL-6 pulse from working muscle exerts anti-inflammatory systemic effects, attenuating hepatic and vascular inflammatory tone through a mechanism that has no pharmacological equivalent [13].

Beyond skeletal muscle, exercise remodels adipose tissue directly. Repeated bouts of aerobic exercise increase capillary density within subcutaneous adipose tissue, improve oxygen delivery and reduce the local hypoxia that drives hypoxia-inducible factor 1α (HIF-1α)-dependent fibrosis and NLRP3 priming. Resistance training additionally preserves skeletal muscle mass, maintaining the principal peripheral glucose disposal mechanism and reducing the glucotoxic burden on pancreatic β-cells [13].

A network meta-analysis by Wang et al. (2025) [13] comparing aerobic, resistance and combined exercise in individuals with overweight and obesity demonstrated that combined training exerted the strongest immunometabolic effect, leading to a significant reduction in circulating leptin and IL-6 concentrations and an increase in adiponectin. At the molecular level, aerobic training promotes angiogenesis in adipose tissue (vascular endothelial growth factor (VEGF)-dependent), reducing hypoxia and HIF-1α activation, a key trigger of NLRP3 priming in adipocytes. Resistance training acts through a different mechanism, directly reducing intramyocellular triglyceride (IMTG) content and ceramide accumulation in myocytes, thereby reducing the principal lipotoxic NLRP3 activation signals in skeletal muscle. Both forms of training promote M1 → M2 polarisation of adipose tissue macrophages through peroxisome proliferator-activated receptor γ (PPARγ) pathway activation, and combined training achieves regression of crown-like structures even in the absence of weight reduction, confirming the immunometabolic benefit independent of caloric balance [14].

### 2.3. Bariatric Surgery

Bariatric surgery represents one of the most comprehensive currently available interventions for modifying multiple components of obesity-associated immunometabolic dysfunction, including adipose tissue biology, intestinal microbiome composition, hypothalamic energy sensing and systemic inflammatory tone. The speed of metabolic improvement following Roux-en-Y gastric bypass is instructive: type 2 diabetes remission may occur before substantial weight loss, supporting an important role for early gut–brain–adipose axis reprogramming through changes in incretin secretion, bile acid signalling and microbiome composition. Adiponectin increases and leptin decreases within weeks, while visceral adipose tissue inflammation and NLRP3-related activity may also be attenuated; together, these changes illustrate the broad network-level effects associated with bariatric surgery [15,16].

These effects are not explained by anatomical restriction alone. The early metabolic improvement observed after surgery, which can precede substantial weight loss, supports a contribution from gut–brain–adipose axis reprogramming, with subsequent structural fat loss further amplifying metabolic benefits [15,16].

Bariatric surgery, based on available data, produces substantial and durable improvements in several markers of adipose tissue immunometabolic dysfunction. A meta-analysis by Huang et al. [15] encompassing 95 studies (*n* = 6232 patients) confirmed significant reductions in leptin, TNF-α and IL-6 concentrations alongside an increase in adiponectin following both Roux-en-Y gastric bypass (RYGB) and sleeve gastrectomy. These effects may reflect not only weight loss but also changes in incretin secretion (glucagon-like peptide-1 (GLP-1), peptide YY (PYY)), gut microbiota composition and SCFA production [15]. Moreover, a study by Palomäki et al. [16] demonstrated directly in adipose tissue biopsies that bariatric surgery was associated with regression of the NLRP3–caspase-1 axis in crown-like structures, providing direct evidence that bariatric surgery can attenuate this inflammatory pathway within human adipose tissue [16].

### 2.4. Sleep and Circadian Rhythm

Sleep deprivation and circadian misalignment are rarely framed as inflammatory interventions, yet their molecular consequences are directly relevant to the network described in this series. Shortened sleep duration below six hours increases cortisol and sympathetic tone, drives free fatty acid (FFA) release from adipose tissue and raises circulating levels of the NLRP3 priming signals IL-6 and TNF-α within 24–48 h. Disrupted circadian rhythmicity additionally impairs the physiological nocturnal suppression of NF-κB activity that normally limits basal inflammatory tone. In the context of the Prometheus’s Fire framework, poor sleep acts as a continuous low-level NLRP3 primer that undermines the efficacy of all other network-targeting interventions, a point that is often overlooked when evaluating the variable response to pharmacotherapy in clinical practice [17,18,19,20].

A meta-analysis by Irwin et al. encompassing 72 studies (*n* > 50,000) confirmed that shortened sleep duration below 6 h is associated with elevated concentrations of IL-6, TNF-α and hs-CRP and a reduction in adiponectin, changes consistent with the inflammatory signature of adipose tissue dysfunction [18]. At the molecular level, sleep deprivation activates NF-κB and the NLRP3 inflammasome through cortisol-mediated suppression of adiponectin expression, augmented sympathetic nervous system activity and elevated circulating free fatty acid concentrations [19]. Circadian rhythm dysregulation, manifested as disrupted *CLOCK*/*BMAL1* gene expression in adipocytes, additionally impairs the chronobiological regulation of lipolysis and adipokine secretion, leading to an unfavourable shift in the adiponectin/leptin ratio and augmented inflammatory signalling throughout the 24 h cycle. A recent review by Tanasescu et al. highlighted adipose tissue circadian dysregulation as a potential contributor to cardiometabolic and cardiovascular risk beyond BMI [20].

### 2.5. Common Biological Targets of Lifestyle Interventions

Despite their apparent mechanistic diversity, non-pharmacological interventions converge on a surprisingly limited set of conserved biological targets within the immunometabolic network. Table 1 summarises the principal molecular targets of lifestyle-based interventions and the specific mechanisms through which caloric restriction, dietary modification, exercise and bariatric surgery engage each. This convergence is not coincidental: it reflects the fact that multiple effective lifestyle interventions reduce lipotoxic substrate burden, restore mitochondrial quality control or improve adipose tissue vascular supply, the three upstream conditions that enable the NLRP3 inflammasome to return to its quiescent state [1,2,3,4,5,11,12,13,14,15,16,18,19,20]. Table 1 provides a mechanistic summary of these shared targets across the four principal lifestyle-based interventions.

Despite their apparent diversity, multiple effective lifestyle interventions converge on a limited number of conserved biological mechanisms. Restoration of AMPK activity, attenuation of NLRP3-dependent inflammasome activation, changes in macrophage inflammatory phenotype and improvement of adiponectin signalling represent recurring biological features of effective lifestyle-based therapy, a pattern that mirrors the therapeutic convergence observed across pharmacological agents and is directly traceable to the molecular network described in Parts I and II of this series. Viewed through this lens, the immunometabolic network described in those earlier papers is not irreversibly established: it may be progressively attenuated by interventions targeting several of its conserved molecular and metabolic components, and lifestyle interventions represent the first and most accessible means of doing so.

## 3. Pharmacological Interventions

A literature search was conducted in PubMed, Embase and the Cochrane Library for publications from January 2019 to August 2026, with the aim of capturing recent clinical and mechanistic evidence. Older seminal and landmark references published before 2019 were included selectively where they represent foundational evidence for the mechanisms or interventions discussed. The inclusion and exclusion criteria were defined before the search. The pharmacological interventions discussed in this section were selected based on two complementary considerations: their established clinical use in obesity-related metabolic disease and the availability of evidence for immunometabolic effects relevant to the biological framework developed in Parts I and II of the Prometheus’s Fire series.

Interventions were considered eligible when peer-reviewed evidence from mechanistic studies, randomised controlled trials or meta-analyses indicated an effect on at least one of the following processes: NLRP3 inflammasome activation, NF-κB-dependent inflammatory signalling, adipokine balance or macrophage phenotype in adipose tissue or peripheral organs.

This approach intentionally includes pharmacological classes with distinct primary mechanisms of action, including biguanides (metformin), sodium–glucose cotransporter-2 (SGLT2) inhibitors, peroxisome proliferator-activated receptor γ (PPARγ) agonists, statins, omega-3 fatty acids, dipeptidyl peptidase-4 (DPP-4) inhibitors and incretin-based therapies. The aim was not to provide an exhaustive overview of obesity pharmacotherapy but rather to examine whether immunometabolic effects observed across pharmacologically diverse interventions converge on biological processes implicated in obesity-associated inflammation. Agents for which sufficient evidence of relevant immunometabolic effects in the context of obesity was not available were therefore not included.

Several pharmacological options used in obesity management were deliberately excluded because they did not meet the predefined mechanistic criteria. Orlistat, a pancreatic lipase inhibitor, reduces dietary fat absorption and produces weight loss; however, evidence for a clinically relevant immunometabolic effect independent of weight reduction remains limited. Naltrexone–bupropion acts centrally through hypothalamic pathways, and although low-dose naltrexone has been reported to attenuate TLR4-dependent NF-κB signalling in preclinical models, clinical evidence for a weight-independent immunometabolic benefit in obesity remains insufficient. Phentermine–topiramate was also excluded because, despite preclinical evidence that topiramate may influence NLRP3 assembly, clinical evidence linking this mechanism to immunometabolic outcomes in obesity remains lacking.

### 3.1. Metformin

Metformin has been used in clinical practice for over six decades, yet its mechanism of action in adipose tissue remains underappreciated. AMPK activation by metformin suppresses TXNIP expression in adipocytes, reducing the availability of the principal intracellular trigger of NLRP3 assembly. Simultaneously, improved mitochondrial efficiency reduces the ROS burden that drives caspase-1 activation downstream of the inflammasome. The resulting attenuation of IL-1β and IL-18 maturation within adipose tissue is modest compared with that produced by incretin-based therapies, but it is mechanistically coherent and explains the anti-inflammatory effects of metformin that have been observed consistently across intervention studies irrespective of glycaemic improvement [21,22,23,24].

The most recent meta-analysis by Zhao et al. (2024), including 49 RCTs, found that metformin significantly reduced circulating TNF-α, IL-6, leptin and resistin levels [21]. Importantly, these effects appeared to be only partly explained by changes in body weight and glycaemic control.

At the molecular level, several mechanisms may contribute to the anti-inflammatory effects of metformin in adipose tissue. Inhibition of mitochondrial complex I activates AMPK, which in turn suppresses mechanistic target of rapamycin complex 1 (mTORC1) signalling and may reduce ROS generation. This could limit oxidative stress-dependent activation of the NLRP3 inflammasome in adipocytes [22,23]. Metformin may also attenuate NF-κB signalling through AMPK-dependent mechanisms involving inhibitor of nuclear factor kappa B alpha (IκBα) and reduce lipopolysaccharide-induced M1 macrophage polarisation. Together, these effects may contribute to the attenuation of adipose tissue inflammation [22,24].

### 3.2. SGLT2 Inhibitors

The cardiovascular and renal benefits of sodium–glucose cotransporter-2 (SGLT2) inhibitors extend beyond their glucose-lowering effects. By reducing intracellular glucose overload and promoting mild ketonaemia, SGLT2 inhibitors may improve mitochondrial energetic efficiency and decrease the metabolic substrate burden that contributes to NLRP3 inflammasome priming. The consequent attenuation of adipose tissue and renal inflammatory signalling, combined with direct haemodynamic effects, may contribute to reduced inter-organ metabolic and inflammatory coupling between visceral adipose tissue, the kidney and the cardiovascular system [25,26,27,28].

A meta-analysis by Wang et al. [25], including 34 RCTs and 6261 participants, found that SGLT2 inhibitors significantly reduced hs-CRP, IL-6, TNF-α and leptin levels while increasing adiponectin concentrations. Importantly, these effects appeared to be only partly explained by improvements in glycaemic control and body weight reduction [25].

One proposed mechanism involves the increase in circulating β-hydroxybutyrate (β-HB) resulting from enhanced ketogenesis during SGLT2 inhibitor therapy. β-HB may suppress NLRP3 inflammasome activity through several complementary mechanisms, including signalling via the hydroxycarboxylic acid receptor 2 (HCAR2) expressed on macrophages and adipocytes [26,27,28]. It has also been reported to interfere with TXNIP-dependent NLRP3 activation by limiting TXNIP dissociation from thioredoxin, thereby attenuating an important upstream signal for inflammasome activation [26,27,28].

Consistent with these observations, a meta-analysis by Liu et al. focusing specifically on adipose tissue reported a significant reduction in visceral adipose tissue (VAT) volume together with improvements in the adipokine profile during SGLT2 inhibitor treatment [26]. These changes may extend beyond those expected from body weight reduction alone, supporting the possibility that SGLT2 inhibitors influence adipose tissue immunometabolism through mechanisms that are not solely attributable to weight loss.

Beyond their effects on adipokines and inflammasome activity, SGLT2 inhibitors have been associated with a shift in macrophage polarisation within adipose tissue. Preclinical evidence from high-fat diet mouse models demonstrates that treatment with ipragliflozin and empagliflozin reduces the ratio of pro-inflammatory M1-like to anti-inflammatory M2-like macrophages in adipose tissue, attenuates crown-like structure formation, and lowers expression of M1-associated markers, including TNF-α and IL-1β, while increasing M2-associated markers, such as CD206 and IL-10 [29,30].

One proposed mechanism linking SGLT2 inhibition to macrophage reprogramming involves the increase in circulating β-hydroxybutyrate (β-HB). In addition to suppressing NLRP3 inflammasome activity through HCAR2 receptor signalling, β-HB has been reported to attenuate IL-15 induction in M1-polarised macrophages, with downstream effects on adipocyte lipogenesis and adipose tissue expansion. This ketone-mediated mechanism may therefore represent an additional pathway through which SGLT2 inhibitors influence the balance of inflammatory macrophage phenotypes within adipose tissue, complementing their established effects on NLRP3 inflammasome activity and NF-κB-dependent signalling [29,30]. It should be noted, however, that direct evidence for SGLT2 inhibitor-induced macrophage polarisation in human adipose tissue remains limited, and current evidence derives predominantly from experimental models.

### 3.3. PPARγ Agonists (Thiazolidinediones)

Thiazolidinediones occupy a distinctive position in obesity pharmacotherapy because they directly target impaired adipose tissue expandability, which was described in Part I as an important initiating event in immunometabolic dysfunction. By activating the nuclear receptor PPARγ, the master regulator of adipogenesis, pioglitazone promotes the differentiation of preadipocytes into smaller, metabolically competent adipocytes. This may partially restore the capacity of adipose tissue to expand through hyperplasia rather than continued hypertrophy [7,31].

The clinical evidence for thiazolidinediones derives primarily from trials in patients with type 2 diabetes and concomitant obesity or metabolic syndrome, populations in which the anti-inflammatory and adiponectin-raising effects of pioglitazone have been most consistently demonstrated [31].

PPARγ activation also suppresses NF-κB signalling and reduces the expression of pro-inflammatory mediators, including TNF-α, IL-6 and monocyte chemoattractant protein-1 (MCP-1), while increasing adiponectin production. By promoting the formation of new adipocytes, thiazolidinediones may improve the capacity of adipose tissue to safely store excess lipids and thereby reduce the lipotoxic stress associated with hypertrophic adipocytes and NLRP3 activation [7,31].

An apparent clinical paradox accompanies these effects: the strong adiponectin raising and adipose tissue remodelling effects of thiazolidinediones occur alongside weight gain. This increase in body weight may partly reflect redistribution of lipid from visceral and other dysfunctional depots towards subcutaneous adipose tissue rather than a simple increase in total lipid burden [31]. However, the potential immunometabolic benefits of thiazolidinediones must be balanced against their adverse effect profile. Peripheral oedema and weight gain can limit their clinical use, while concerns regarding cardiovascular safety, particularly with rosiglitazone, have further restricted their role in contemporary obesity management [7,31].

### 3.4. Statins

Statins are not obesity treatments, and their role as network-targeting agents therefore requires some qualification. Their pleiotropic anti-inflammatory effects are well documented but are generally more modest than those of interventions that directly target the metabolic and inflammatory drivers of obesity. In the context of this series, their relevance lies mainly in the modulation of inter-organ inflammatory signalling. By improving endothelial function and reducing vascular inflammatory tone, statins may partially attenuate the perivascular adipose tissue (PVAT), endothelium and atherosclerotic plaque loop described in Part II. This may be clinically relevant in patients with obesity and established cardiovascular disease, even if their effects are not primarily directed at adipose tissue [32].

A meta-analysis by Jain and Ridker including 30 randomised trials and more than 10,000 participants reported significant reductions in hs-CRP and IL-6 concentrations, together with improvements in endothelial function, that were not fully explained by the extent of low-density lipoprotein cholesterol (LDL-C) reduction [32]. Several mechanisms may contribute to these effects. By reducing Rho and Rac GTPase prenylation, statins may decrease nicotinamide adenine dinucleotide phosphate (NADPH) oxidase-dependent ROS production in adipocytes and macrophages and thereby limit NLRP3 inflammasome activation. They may also attenuate NF-κB signalling by reducing the availability of geranylgeranyl pyrophosphate (GGPP), an intermediate of the mevalonate pathway involved in inflammatory signalling. Within adipose tissue, these effects may contribute to reduced M1 macrophage polarisation and fewer crown-like structures, potentially supporting local immunometabolic homeostasis [32].

### 3.5. Omega-3 Fatty Acids

Long-chain omega-3 fatty acids, particularly eicosapentaenoic acid (EPA) and docosahexaenoic acid (DHA), occupy a somewhat different position within the immunometabolic network than the other interventions discussed above. Rather than primarily suppressing inflammatory signalling, they may help shift the inflammatory response towards resolution. EPA and DHA can replace arachidonic acid in membrane phospholipids and serve as precursors for specialised pro-resolving mediators, including resolvins, protectins and maresins. These mediators actively promote the return of tissues to homeostasis rather than simply reducing the intensity of inflammatory signalling [33].

Several complementary mechanisms may contribute to these effects. EPA and DHA can activate GPR120 on macrophages and adipocytes, engaging β arrestin 2 signalling and attenuating inhibitor of nuclear factor kappa B kinase subunit beta (IKKβ) and NF κB activation. This may reduce inflammatory priming of the NLRP3 inflammasome in adipose tissue macrophages [33]. EPA and DHA also serve as substrates for the formation of E-series and D-series resolvins and maresins. Among these mediators, maresin 1 has been reported to attenuate caspase-1 activation and reduce the release of IL-1β and IL-18, suggesting a potential role in limiting inflammasome-driven inflammation. DHA is also a precursor of neuroprotectin D1, which may further support resolution by reducing the production of pro-inflammatory lipid mediators and promoting a more reparative macrophage phenotype [33].

This distinction between inflammatory suppression and resolution may be particularly relevant to the adipose tissue network. Omega 3 supplementation may be less important as a primary means of suppressing NLRP3 activation and more relevant as a mechanism that facilitates the resolution of inflammation after its initial drivers have been addressed. Emerging evidence from 2022 to 2024 also suggests that the magnitude of these effects may depend on the EPA-to-DHA composition of the preparation and on the baseline inflammatory state. Greater responses have been reported in individuals with higher baseline concentrations of inflammatory markers, such as IL-6 and TNF-α, raising the possibility that inflammatory profiling could eventually help identify patients most likely to benefit from this approach [33].

### 3.6. DPP-4 Inhibitors

Dipeptidyl peptidase 4 inhibitors (DPP-4i, gliptins) provide an instructive counterexample to the concept of therapeutic network engagement. By inhibiting the enzyme responsible for the degradation of endogenous GLP-1 and glucose-dependent insulinotropic polypeptide (GIP), these agents prolong incretin activity and might therefore be expected to reproduce at least some of the anti-inflammatory effects observed with direct incretin receptor agonists. However, their clinical effects have generally been less pronounced. The cardiovascular outcome trials SAVOR TIMI, TECOS and EXAMINE did not demonstrate the broader cardiovascular benefits associated with GLP-1 receptor agonists or SGLT2 inhibitors [34,35].

Several factors may help explain this difference. DPP-4 inhibitors produce a relatively modest increase in circulating endogenous GLP-1, generally around two to three-fold, compared with the substantially greater exposure achieved with pharmacological GLP-1 receptor agonists. This difference in the magnitude and duration of receptor stimulation may limit the extent to which endocrine incretin signalling translates into tissue-level effects within adipose tissue and other organs [34,35]. In this context, DPP-4 inhibitors illustrate an important principle of the immunometabolic network: engaging a pathway does not necessarily produce the same biological consequences as sustained and more intensive activation of that pathway.

DPP-4 inhibition may nevertheless influence adipose tissue inflammation through mechanisms that extend beyond incretin signalling. Adipose tissue is an important source of DPP-4, and increased DPP-4 secretion by hypertrophic adipocytes has been associated with local inflammation. DPP-4 has also been proposed to act as a pro-inflammatory adipokine, with effects on TLR4 and NF-κB signalling in adipose tissue macrophages [34,36]. Inhibition of DPP-4 may therefore reduce the activity of this pathway while simultaneously prolonging endogenous incretin action. A meta-analysis by Makdissi et al., including 26 RCTs and 2748 participants, reported a significant reduction in hs-CRP and an increase in adiponectin, although these effects were less pronounced than those reported with direct GLP-1 receptor agonism [34,36].

Overall, these findings suggest that the immunometabolic effects of DPP-4 inhibitors are likely to depend on both incretin-dependent and incretin-independent mechanisms. Their relatively modest effects also highlight that partial engagement of a network node may not be sufficient to produce the broader systemic effects associated with more sustained pharmacological activation [34,35,36].

### 3.7. GLP-1 Receptor Agonists and Dual GLP-1R/GIPR Agonist (Tirzepatide)

GLP-1 receptor agonists are currently the only pharmacological class for which robust clinical evidence supports effects across several of the therapeutic nodes considered in this review. Their actions extend beyond weight loss and include modulation of inflammasome activity, adipokine signalling, adipose tissue remodelling and inter-organ inflammatory communication. This broader biological activity may help explain the consistent multi-organ benefits observed across major clinical trials, including LEADER, SUSTAIN 6, SELECT and FLOW [37,38,39,40,41,42,43,44,45,46,47].

Tirzepatide adds another dimension through simultaneous activation of GIP receptor (GIPR) and GLP-1 receptor (GLP-1R). GIPR is expressed in adipose tissue, where its activation influences lipid handling, adipogenesis and thermogenic pathways. These effects may contribute to improved adipose tissue expandability and metabolic function through mechanisms that are not fully reproduced by GLP-1R agonism alone [37,38,48,49].

#### 3.7.1. Anti-Inflammatory Effects on Adipose Tissue

GLP-1 receptor agonists and tirzepatide have been associated with multiple immunometabolic effects in adipose tissue. Their effects extend beyond reductions in adipose tissue mass and circulating free fatty acids and may involve changes in adipocytes, adipose tissue macrophages and the systemic adipokine profile [37,38]. Systematic reviews and meta-analyses published between 2021 and 2026 have reported reductions in hs CRP, IL-6, TNF-α and leptin, together with increases in adiponectin following GLP-1 receptor agonist treatment [37,38].

Several mechanisms may contribute to these effects. GLP-1R signalling can influence NF-κB and NLRP3 pathways, while cyclic adenosine monophosphate (cAMP) and protein kinase A (PKA) signalling may modify TXNIP activity and thereby reduce an important stimulus for inflammasome activation. GLP-1 receptor agonism may also enhance AMPK and autophagy-related pathways, potentially improving mitochondrial quality and limiting the generation of reactive oxygen species involved in NLRP3 activation [37].

Changes in macrophage phenotype provide another possible component of this response. Preclinical studies and transcriptomic analyses suggest that GLP-1 signalling can reduce adipose tissue macrophage infiltration and shift inflammatory macrophage-related gene expression towards a less pro-inflammatory profile and favour a more reparative phenotype. These changes have been associated with fewer crown-like structures and improved local adipose tissue homeostasis [37,50]. The relative contribution of direct receptor signalling and indirect effects related to weight loss, however, remains an important area of investigation.

#### 3.7.2. GIPR-Mediated Effects on Adipose Tissue Metabolism

One feature that distinguishes tirzepatide from GLP-1 receptor agonist monotherapy is its additional activity at the GIP receptor. GIPR is expressed in adipose tissue, including visceral and subcutaneous depots, and its activation can influence adipocyte metabolism and differentiation. The extent and functional relevance of GLP-1R expression in human adipocytes, in contrast, remain less clearly established [48,49].

GIPR signalling in adipocytes has been linked to changes in lipid uptake, adipogenesis and adipokine secretion, including effects on adiponectin and leptin. These mechanisms may complement the metabolic and inflammatory effects associated with GLP-1R signalling in adipose tissue and peripheral organs [48,49].

Chronic GIPR activation has also been associated with improved insulin signalling in adipocytes, changes in macrophage infiltration and increased thermogenic activity in brown adipose tissue. Experimental studies suggest that calcium cycling through sarco/endoplasmic reticulum Ca^2+^-ATPase (SERCA) may contribute to this response [51]. In white adipose tissue, changes in lipid turnover and storage could reduce the accumulation of potentially lipotoxic metabolites, including ceramides and diacylglycerols, which have been implicated in inflammasome activation [48,51,52].

Recent meta-analyses have reported greater reductions in visceral adipose tissue and more favourable changes in adiponectin, leptin and selected inflammatory markers with tirzepatide than with GLP-1 receptor agonist monotherapy in comparable treatment settings [53]. These findings support the possibility that dual receptor activation influences adipose tissue biology in ways that extend beyond the effect of weight loss alone.

The reduction in visceral adipose tissue observed with tirzepatide may therefore represent more than a simple consequence of reduced energy intake. The SURPASS-3 MRI substudy demonstrated significant reductions in liver fat content and abdominal visceral adipose tissue with tirzepatide [54]. A subsequent analysis of the SURPASS-3 MRI cohort also demonstrated favourable changes in muscle composition and reductions in intramuscular fat [43]. These findings are compatible with improved lipid partitioning and reduced lipid delivery to non-adipose tissues, although the extent to which they reflect direct tissue actions rather than secondary effects of weight loss remains uncertain.

#### 3.7.3. Beyond Adipose Tissue: Organ-Specific and Cardiometabolic Effects

Among GLP-1 receptor agonists, liraglutide provided some of the early evidence linking changes in adipose tissue inflammation with broader clinical outcomes. Experimental and translational studies have associated liraglutide with reduced macrophage infiltration, fewer crown-like structures and lower TNF-α expression in adipose tissue, together with increased adiponectin [37,45]. In LEADER, liraglutide was also associated with a significant reduction in major adverse cardiovascular events [45].

Semaglutide has provided further evidence for broader cardiometabolic effects. Clinical and meta-analytic data indicate reductions in hs-CRP and other inflammatory markers, together with favourable changes in adipokines [35,37,46]. Some of these effects have remained detectable after adjustment for weight loss, supporting the possibility of biological actions that are not fully explained by reduced adiposity.

Imaging studies provide additional evidence for effects on vascular adipose tissue. Semaglutide has been associated with reductions in coronary fat attenuation index, an imaging marker related to perivascular adipose tissue inflammation, with changes that may not be fully explained by glycaemic improvement or weight loss [38,47]. GLP-1 receptor agonists have also been associated with reductions in epicardial adipose tissue volume across randomised trials [35,37,47].

At the class level, analyses of randomised trials suggest that GLP-1 receptor agonists reduce visceral adipose tissue and may favourably influence macrophage infiltration, crown-like structures and adiponectin concentrations. The persistence of some effects after adjustment for weight loss supports a pharmacological contribution, although the relative importance of direct tissue actions and secondary effects of weight reduction remains difficult to determine [37,38].

The effects of incretin-based therapies also extend to the liver. In the ESSENCE trial, semaglutide 2.4 mg was associated with metabolic dysfunction-associated steatohepatitis (MASH) resolution in 62.9% of participants compared with 34.3% receiving placebo, while improvement in fibrosis occurred in 36.8% and 22.4%, respectively [39]. In the SYNERGY-NASH trial, tirzepatide achieved MASH resolution in 44%, 56% and 62% of participants receiving the 5 mg, 10 mg and 15 mg doses respectively, compared with 10% in the placebo group. Improvement of at least one fibrosis stage was observed in 55%, 51% and 51% of participants in the respective dose groups, compared with 30% with placebo [40].

The SELECT trial demonstrated the cardiovascular relevance of this therapeutic class. Semaglutide reduced major adverse cardiovascular events by 20% in patients with obesity and established cardiovascular disease without diabetes [35]. The magnitude of this benefit cannot be attributed to weight loss alone with certainty, and several mechanisms may contribute, including improved metabolic control, reduced inflammation and changes in ectopic and vascular adipose tissue.

In patients with obesity-related heart failure with preserved ejection fraction (HFpEF), the STEP-HFpEF trial demonstrated significant improvements in symptoms, physical limitations and exercise function with semaglutide, together with reductions in N-terminal pro-B-type natriuretic peptide (NT-proBNP) and body weight [55]. Reductions in epicardial adipose tissue may represent one component of this response, although the relative contribution of cardiac adipose tissue and other systemic mechanisms remains uncertain.

Evidence for effects on pancreatic β-cells adds another dimension. A meta-analysis of 16 RCTs including 6591 participants found that semaglutide significantly improved homeostatic model assessment of β-cell function (HOMA-B), with the greatest effects observed in patients with a shorter duration of type 2 diabetes mellitus (T2DM) [44]. These findings raise the possibility of a clinically relevant window in which preservation of β cell function may be more achievable. Experimental data also suggest complementary effects of GIPR and GLP-1R activation on β cell survival and function, although the extent to which these mechanisms translate into long-term preservation in humans remains to be established.

#### 3.7.4. Tirzepatide: Integrated Metabolic and Inflammatory Effects

The SURMOUNT 5 trial provided the first direct head-to-head comparison of tirzepatide and semaglutide in adults with obesity without T2DM. Tirzepatide produced greater weight reduction than semaglutide, 20.2% versus 13.7%, respectively, and was also associated with favourable changes in visceral adipose tissue and selected metabolic and inflammatory markers [56].

The cardiovascular evidence is also evolving. In SURPASS-CVOT, tirzepatide demonstrated cardiovascular safety compared with dulaglutide [42]. Separately, tirzepatide improved clinical outcomes in patients with obesity-related HFpEF in the SUMMIT trial [41]. These findings, together with results from obesity and liver disease trials, are consistent with effects that extend beyond body weight reduction.

From the perspective developed in this review, the significance of tirzepatide therefore lies not only in the magnitude of weight loss it produces but also in the range of biological processes influenced by simultaneous GIPR and GLP-1R activation. Its effects on adipose tissue, liver, cardiovascular system, kidney and pancreatic β-cells provide a clinically relevant example of how modulation of interconnected pathways may translate into benefits across multiple organs.

At the same time, an important distinction should be maintained between association and mechanism. Some observed benefits may result directly from receptor-mediated effects, whereas others may arise secondarily from weight loss, improved glycaemic control or changes in substrate availability. Disentangling these contributions will be important for determining which components of the immunometabolic network are most responsive to dual incretin therapy and whether these effects can be maintained over the long-term.

Overall, evidence from recent meta-analyses and large clinical trials indicates that the effects of incretin-based therapies extend beyond simple reduction of adipose tissue mass and encompass several interconnected aspects of adipose tissue immunometabolism [50,54,55,57]. GLP-1 receptor agonists and tirzepatide show particularly broad effects, including attenuation of inflammatory signalling, improvement of adipokine balance, modulation of macrophage activity and reduction of pathological adipose tissue–organ communication. The clinical benefits associated with these changes extend across cardiovascular, hepatic and metabolic outcomes, although the relative contribution of individual mechanisms remains difficult to disentangle from the effects of weight loss.

More broadly, the pharmacological interventions reviewed in this section illustrate a recurring principle of obesity treatment: different therapeutic entry points can converge on overlapping biological processes that sustain chronic immunometabolic dysfunction. Depending on their primary mechanism, individual interventions may influence inflammatory signalling, mitochondrial stress, adipokine secretion, macrophage phenotype, adipose tissue remodelling or inter-organ metabolic communication to different degrees. Rather than representing independent therapeutic targets, these processes are closely interconnected and may reinforce one another within the self-amplifying inflammatory network described in Parts I and II.

This convergence provides a useful framework for comparing pharmacological strategies without reducing their effects to a fixed number of therapeutic nodes. Table 2 therefore summarises the principal biological pathways and processes influenced by the pharmacological interventions reviewed in this section, highlighting both their shared effects and their mechanistic differences.

### 3.8. Common Biological Targets of Pharmacological Interventions

Pharmacological interventions also converge on a limited set of conserved biological targets, although their effects differ in magnitude, specificity and the extent to which they influence adipose tissue immunometabolism. The interventions reviewed in this section influence several interconnected processes, including NLRP3 inflammasome activity, NF-κB signalling, adipokine balance, macrophage phenotype, mitochondrial function, adipose tissue fibrosis and tissue expandability. Across these interventions, the available evidence suggests overlapping effects on several biological processes rather than a uniform mechanism of action [6,7,8,21,22,23,24,25,26,27,28,31,32,33,34,35,37,38,39,40,41,42,43,44,45,46,47,48,49,50,51,52,53,54,55,56,57]. Table 2 provides a mechanistic overview of these shared biological targets and the principal pathways through which individual pharmacological classes may influence them.

## 4. Convergence of Pharmacological and Non-Pharmacological Interventions

Despite their different modes of action, pharmacological and non-pharmacological interventions converge on a limited number of conserved biological targets within the immunometabolic network. As summarised in Table 1 and Table 2, both intervention categories influence NLRP3 inflammasome activity, NF-κB signalling, adipokine balance, macrophage phenotype, mitochondrial quality control, adipose tissue fibrosis and tissue expandability. Thus, their apparent therapeutic diversity masks a substantial degree of biological convergence. The main distinction lies not in the targets themselves, but in the extent, specificity and breadth with which individual interventions influence these pathways [1,2,3,21,25,37,38].

The principal molecular targets engaged by the pharmacological interventions reviewed in this part are summarised in Figure 1. Rather than acting through isolated pathways, these mechanisms are interconnected, such that modulation of one process may influence several downstream components of adipose tissue dysfunction. NLRP3 inflammasome and NF-κB signalling represent major inflammatory mechanisms, while the adiponectin–leptin axis, macrophage phenotype and mitochondrial function link inflammatory activity with the metabolic function of adipose tissue [21,22,25,37,38,48,50].

### Comparative View of Therapeutic Interventions

Table 1 and Table 2 and Figure 1 indicate that effective interventions do not act through a single inflammatory pathway. Rather, they progressively modify the interconnected processes that maintain adipose tissue dysfunction. Lifestyle-based interventions primarily reduce the upstream metabolic pressures that initiate and sustain inflammation, including excess substrate availability, mitochondrial stress, adipocyte hypertrophy and impaired tissue perfusion. Pharmacological interventions, in contrast, can engage selected molecular nodes more directly, although the extent of network modulation differs substantially between drug classes [1,2,8,13,21,37].

This distinction is particularly evident when the two intervention categories are considered side by side. Caloric restriction, physical activity, dietary modification and bariatric surgery primarily act by reducing metabolic and mechanical stress on adipose tissue and by improving its capacity to safely store and process lipids. Pharmacological agents may additionally target specific signalling pathways, such as AMPK, PPARγ, incretin receptors or inflammatory signalling cascades. The resulting effects therefore overlap biologically but differ in the route and intensity through which the network is modulated [8,11,13,23,25,28,37].

Among non-pharmacological interventions, caloric restriction combined with regular physical activity provides the most consistent evidence for improvement of adipose tissue immunometabolism. The Mediterranean diet contributes through several complementary mechanisms, including modulation of NF-κB signalling, gut-derived metabolites and pro-resolving lipid mediators. Bariatric surgery produces the most profound and durable metabolic remodelling, accompanied by substantial reductions in adipose tissue inflammation and improvements in adipose tissue function [5,6,8,11,13,15,16].

Among pharmacological interventions, GLP-1 receptor agonists and tirzepatide show the broadest clinically documented engagement of the immunometabolic network. Their effects extend beyond weight reduction to include improvements in adipose tissue distribution and function, adipokine balance and inflammatory signalling, with downstream effects on cardiovascular, hepatic and renal outcomes. Other pharmacological classes occupy more specific positions within the network. DPP-4 inhibitors provide a useful example of a more limited degree of incretin-mediated network engagement [21,22,23,28,31,33,34,37,38,39,40,41,42,43,44,45,46,47,48,50].

This comparison suggests that therapeutic efficacy may depend less on the complete suppression of any single inflammatory pathway than on the simultaneous attenuation of several mutually reinforcing processes. Interventions that reduce substrate overload, improve mitochondrial quality control, restore adipose tissue expandability and dampen inflammatory signalling may therefore have greater potential to destabilise the self-amplifying loops described in Parts I and II than interventions acting on an isolated downstream mediator [1,2,3,37,38].

This framework also provides a rationale for combining pharmacological and non-pharmacological approaches. Rather than viewing lifestyle modification and pharmacotherapy as competing strategies, they may be considered complementary modes of network modulation: lifestyle interventions predominantly reduce the upstream metabolic conditions that sustain dysfunction, whereas pharmacological therapies can more directly modulate selected molecular hubs. The extent to which these effects are additive or complementary remains an important area for future investigation [1,8,21,37].

Future studies should therefore move beyond body weight as the principal measure of therapeutic response and evaluate qualitative markers of adipose tissue function and immunometabolic remodelling. Identification of biomarkers capable of detecting changes in inflammatory activity, adipokine signalling, mitochondrial stress and adipose tissue phenotype may allow earlier assessment of treatment response and help identify patients who require intensification or combination therapy. Such an approach could ultimately support a more mechanism-based and personalised treatment of obesity and its systemic complications [1,2,25,37].

## 5. Limitations

Several limitations should be considered when interpreting the evidence discussed in Part III. The interventions reviewed here differ considerably in their mechanisms, treatment duration, patient populations and clinical endpoints, which makes direct comparison of their immunometabolic effects difficult. For some interventions, mechanistic evidence comes mainly from experimental or *ex vivo* studies, whereas clinical studies more often rely on circulating inflammatory markers and changes in body composition rather than direct assessment of adipose tissue inflammation. It therefore remains difficult to determine how closely changes in circulating biomarkers reflect local changes within adipose tissue [1,8,21,37].

Another important limitation is the difficulty of distinguishing effects related to weight loss from effects that are specific to the intervention itself. In many clinical studies, improvements in adipokine profiles, inflammatory markers or ectopic fat cannot be clearly separated from the consequences of reduced energy intake and adipose tissue mass. This is particularly relevant when comparing incretin-based therapies with caloric restriction, exercise or bariatric surgery. In addition, evidence for some proposed mechanisms remains heterogeneous, and findings from experimental models do not always translate directly to human adipose tissue biology [8,37,38,39,40].

Finally, the concept of therapeutic convergence presented in this review should be regarded as a mechanistic framework rather than a clinically validated model. Although different interventions influence several overlapping inflammatory and metabolic processes, it remains uncertain whether targeting these processes simultaneously necessarily produces additive clinical effects. Longitudinal studies combining measures of adipose tissue function with validated immunometabolic biomarkers and clinically relevant outcomes will be needed to determine whether this framework can ultimately help identify treatment responders and guide more personalised therapeutic strategies [1,2,3].

## 6. Integrated Conclusions and Future Perspectives

The three parts of the Prometheus’s Fire series describe a coherent mechanistic continuum: Part I proposes impaired adipose tissue expandability and NLRP3 inflammasome activation as key early events; Part II characterises the self-amplifying organ-to-organ inflammatory interactions that may transform local adipose dysfunction into systemic metabolic disease; and Part III examines how diverse therapeutic interventions converge on overlapping immunometabolic mechanisms. Together, these observations provide a biological framework for understanding obesity treatment beyond weight reduction alone [1,2,3].

The evidence reviewed throughout this series supports a transition from weight-centred obesity management toward restoration of adipose tissue immunometabolic quality and attenuation of the self-amplifying inflammatory processes that sustain systemic disease [1,2,37,38].

Despite considerable progress over the past decade, several important questions remain unresolved. Reliable biomarkers that can identify the restoration of adipose tissue immunometabolic function in individual patients have not yet been validated in prospective studies. The optimal sequencing and combination of therapeutic interventions also remain unclear. Equally uncertain are the durability of immunometabolic improvements after treatment discontinuation and the extent to which these improvements can prevent the re-establishment of pro-inflammatory interactions. Future studies should therefore prioritise longitudinal designs that integrate molecular and imaging-based measures of adipose tissue function with conventional clinical endpoints. Where feasible, serial tissue sampling, spatial transcriptomics and metabolomic profiling could provide further mechanistic insight [1,25,37].

The concept of therapeutic convergence may also provide a useful framework for the rational design of combination therapies. Rather than combining interventions solely to maximise weight loss, future strategies could target complementary components of the immunometabolic network. For example, suppression of inflammasome activity could be combined with approaches that restore adipogenic capacity, while interventions targeting mitochondrial homeostasis could complement macrophage reprogramming. The dual GLP-1R/GIPR activity of tirzepatide provides a clinically relevant example of this principle, engaging complementary metabolic pathways that may not be activated to the same extent by single-receptor agonism [38,48,49,50,51,52,56].

This systems-oriented perspective also suggests that the evaluation of therapeutic efficacy should extend beyond body weight and glycaemic control. Functional measures of adipose tissue immunometabolic status, including the adiponectin-to-leptin ratio, circulating ceramides and imaging-derived indicators of adipose tissue inflammation, may provide complementary information about tissue-level responses to treatment. However, their value as biomarkers of therapeutic response remains to be established in prospective studies [25,37].

Importantly, the effects of different interventions cannot always be fully explained by changes in body weight or by a single tissue-specific mechanism. In selected therapeutic settings, improvements in hepatic steatosis, cardiovascular and renal outcomes, and adipose tissue remodelling may exceed or persist beyond those expected from weight reduction alone. These observations are consistent with a broader model in which effective interventions influence several interconnected components of the immunometabolic network, thereby weakening the self-amplifying processes that sustain systemic metabolic dysfunction [35,37,39,40,41,42,55,56].

A central observation emerging from this synthesis is therefore the therapeutic convergence of apparently distinct interventions. Lifestyle modification, bariatric surgery and pharmacological therapies act through different primary mechanisms, yet many ultimately influence overlapping processes, including inflammasome activity, adipokine signalling, mitochondrial homeostasis, macrophage phenotype and adipose tissue remodelling. This convergence does not imply that all interventions are equivalent. Their clinical and biological effects depend on the mechanisms engaged, the magnitude of their effects and the extent to which multiple processes are modified simultaneously [1,2,8,21,25,37,38].

The therapeutic landscape of obesity may therefore be viewed not simply as a collection of interventions targeting individual risk factors but as a set of complementary strategies capable of modifying a shared immunometabolic system. The concept of therapeutic convergence developed throughout the Prometheus’s Fire series provides a framework for understanding why interventions with distinct primary mechanisms may produce overlapping multi-organ benefits. At the same time, it highlights the need to determine which combinations, biomarkers and treatment sequences can most effectively and durably restore metabolic health [1,2,3].

One of the central paradoxes emerging from this synthesis is that weight loss, the most visible marker of therapeutic success, may not always capture the less visible processes that determine the quality and durability of metabolic recovery. This synthesis therefore raises a broader question: should obesity treatment primarily aim to reduce body weight, or should it focus on interrupting the self-sustaining inflammatory processes associated with adipose tissue dysfunction? The evidence reviewed here suggests that these goals should not be viewed as mutually exclusive. Reducing adiposity addresses important upstream drivers of immunometabolic dysfunction, including adipocyte hypertrophy, substrate overload, lipotoxicity and ectopic fat accumulation. Pharmacological interventions may additionally influence specific inflammatory and metabolic processes that contribute to the persistence of dysfunction. From this perspective, the future of obesity therapy may lie not in choosing between weight loss and targeted network modulation, but in combining interventions according to their complementary effects on both upstream drivers and downstream mechanisms of immunometabolic disease. Whether such mechanism-based combinations can provide additive benefits beyond weight reduction alone remains an important question for future prospective trials [1,8,37,38].

## Figures and Tables

**Figure 1:**
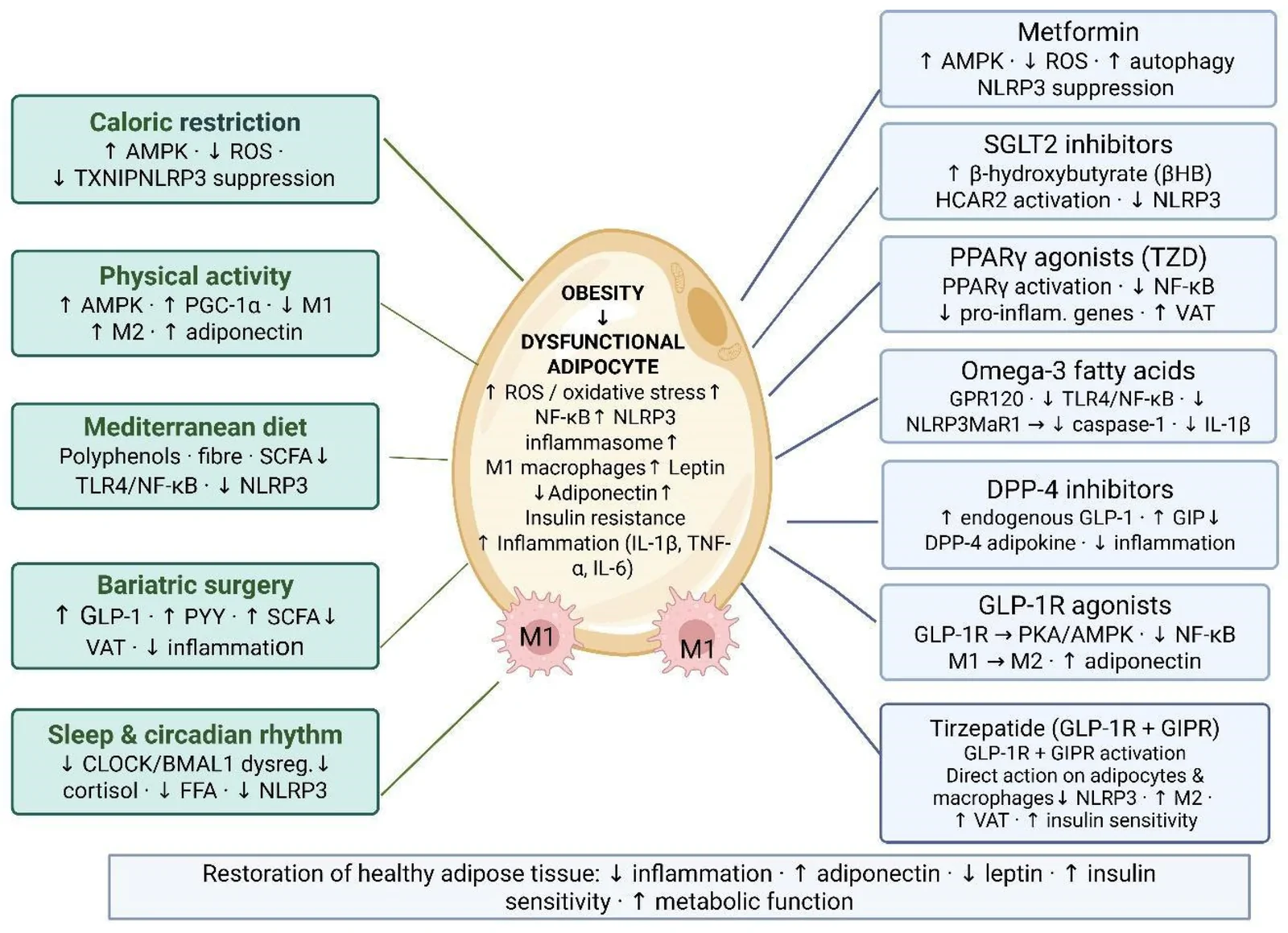
Therapeutic convergence of pharmacological and non-pharmacological interventions on molecular and cellular targets in obesity-associated adipose tissue immunometabolic dysfunction. The diagram illustrates how lifestyle-based, surgical and pharmacological interventions converge on interconnected molecular and cellular processes, including NLRP3 inflammasome activation, NF-κB-dependent inflammatory signalling, adipokine balance, macrophage polarisation and mitochondrial function. Arrows indicate the predominant direction of effect. Created in BioRender (BioRender Inc., Toronto, ON, Canada). Grudniewska, A. (2026) https://BioRender.com/iczw1dn. Abbreviations:. AMPK, AMP-activated protein kinase; βHB, β-hydroxybutyrate; BMAL1, brain and muscle ARNT-like protein 1; CLOCK, circadian locomotor output cycles kaput; DPP-4i, dipeptidyl peptidase-4 inhibitor; FFA, free fatty acids; GIP, glucose-dependent insulinotropic polypeptide; GIPR, GIP receptor; GLP-1, glucagon-like peptide-1; GLP-1R, glucagon-like peptide-1 receptor; GLP-1RA, glucagon-like peptide-1 receptor agonist; GPR120, G protein-coupled receptor 120; HCAR2, hydroxycarboxylic acid receptor 2; IL-1β, interleukin-1β; IL-6, interleukin-6; M1, classically activated macrophage; M2, alternatively activated macrophage; MaR1, Maresin 1; NF-κB, nuclear factor κB; NLRP3, NOD-, LRR- and pyrin domain-containing protein 3; PGC-1α, PPARγ coactivator 1α; PKA, protein kinase A; PPARγ, peroxisome proliferator-activated receptor γ; PYY, peptide YY; ROS, reactive oxygen species; SCFA, short-chain fatty acids; SGLT2i, sodium-glucose cotransporter-2 inhibitor; TLR4, Toll-like receptor 4; TNF-α, tumour necrosis factor α; TXNIP, thioredoxin-interacting protein; TZD, thiazolidinedione; VAT, visceral adipose tissue; ↑, increase or upregulation; ↓, decrease or downregulation.

**Table 1:** Common biological targets of non-pharmacological interventions in obesity-associated immunometabolic disease. Abbreviations: AMPK, AMP-activated protein kinase; ECM, extracellular matrix; FFA, free fatty acid; HIF-1α, hypoxia-inducible factor 1α; IL-6, interleukin-6; NF-κB, nuclear factor κB; NLRP3, NOD-, LRR- and pyrin domain-containing protein 3; PGC-1α, PPARγ coactivator 1α; ROS, reactive oxygen species; SIRT1, sirtuin 1; TGF-β, transforming growth factor β; TNF-α, tumour necrosis factor α; TXNIP, thioredoxin-interacting protein; adipoQ, adiponectin; M1, pro-inflammatory macrophage phenotype; M2, anti-inflammatory macrophage phenotype; PPARγ, peroxisome proliferator-activated receptor γ; ↑, increase or upregulation; ↓, decrease or downregulation.

Biological Target	Lifestyle Interventions and Mechanisms
NLRP3 inflammasome suppression	Caloric restriction (↓ TXNIP, ↓ FFA/ceramide burden); aerobic exercise (↑ AMPK, ↑ mitophagy, ↓ ROS); bariatric surgery (↓ lipotoxic load, gut axis reset)
NF-κB inhibition	Mediterranean diet (polyphenols → SIRT1/AMPK); aerobic exercise (↓ chronic TNF-α/IL-6); bariatric surgery (↓ adipose tissue macrophage infiltration)
Adiponectin restoration	Mediterranean diet (↑ adipoQ via PPARγ/SIRT1); exercise (↑ adipoQ, ↓ visceral fat); bariatric surgery (most marked ↑ within weeks of surgery)
Leptin resistance reversal	Caloric restriction (↓ leptin proportional to fat mass reduction); bariatric surgery (rapid normalisation); sleep optimisation (↓ sympathetic tone, ↓ cortisol-driven leptin secretion)
M1 → M2 macrophage reprogramming	Aerobic exercise (IL-6 pulse → PGC-1α, M2 polarisation); Mediterranean diet (polyphenols, omega-3 → resolvins); bariatric surgery (↓ crown-like structures in adipose tissue)
Mitochondrial dysfunction recovery	Exercise (↑ PGC-1α, mitophagy, biogenesis); caloric restriction (↓ substrate overload, ↑ AMPK-driven efficiency); sleep normalisation (↓ oxidative stress)
Adipose tissue fibrosis	Bariatric surgery (↓ HIF-1α, ↓ TGF-β, ECM remodelling); sustained weight loss ≥ 10% (↓ myofibroblast activation); aerobic exercise (↑ adipose tissue perfusion, ↓ hypoxia-driven fibrosis)
Adipose tissue expandability	Bariatric surgery (depot redistribution, ↑ subcutaneous capacity); caloric restriction (↓ hypertrophic burden, ↓ threshold exceedance); Mediterranean diet (preservation of adipogenic progenitor pools)

**Table 2:** Common biological targets of pharmacological interventions in obesity-associated immunometabolic disease. Pharmacological interventions influence overlapping components of the immunometabolic network, including NLRP3 inflammasome activity, NF-κB signalling, adipokine balance, macrophage phenotype, mitochondrial homeostasis, adipose tissue fibrosis and expandability. Abbreviations: AMPK, AMP-activated protein kinase; GPR120, G protein-coupled receptor 120; NF-κB, nuclear factor κB; NLRP3, NOD-, LRR- and pyrin domain-containing protein 3; PPARγ, peroxisome proliferator-activated receptor γ; SGLT2, sodium-glucose cotransporter 2; cAMP, cyclic adenosine monophosphate; DPP-4, dipeptidyl peptidase-4; GIPR, glucose-dependent insulinotropic polypeptide receptor; GLP-1R, glucagon-like peptide-1 receptor; IKKβ, inhibitor of nuclear factor κB kinase subunit β; IL-1β, interleukin-1β; JNK, c-Jun N-terminal kinase; M1, pro-inflammatory macrophage phenotype; M2, anti-inflammatory macrophage phenotype; PKA, protein kinase A; ROS, reactive oxygen species; TLR, Toll-like receptor; TXNIP, thioredoxin-interacting protein; TZDs, thiazolidinediones; β-HB, β-hydroxybutyrate; β-arrestin-2, beta-arrestin-2; ↑, increase or upregulation; ↓, decrease or downregulation.

Biological Target	Intervention	Mechanism
NLRP3 inflammasome suppression	Metformin	↑ AMPK, ↓ mitochondrial ROS, ↓ TXNIP/NLRP3 signalling
	SGLT2 inhibitors	Improved metabolic substrate handling, ↓ oxidative stress and inflammatory signalling
	GLP-1 receptor agonists	↑ cAMP/PKA, ↑ AMPK/autophagy, ↓ NF-κB priming and NLRP3 activation
	Tirzepatide	Combined GLP-1R/GIPR signalling, ↓ lipotoxicity and inflammatory activation
	Statins	↓ Oxidative stress and NLRP3-related signalling
	Omega-3 fatty acids	GPR120/β-arrestin-2 signalling and specialised pro-resolving mediators
NF-κB inhibition	Metformin	AMPK-dependent inhibition of NF-κB signalling
	PPARγ agonists/TZDs	PPARγ-mediated transrepression of inflammatory genes
	GLP-1 receptor agonists	↓ NF-κB activation and pro-inflammatory cytokine transcription
	Tirzepatide	Combined incretin signalling with reduced inflammatory tone
	Statins	↓ Rho/Rac-dependent inflammatory signalling
	Omega-3 fatty acids	GPR120/β-arrestin-2-mediated inhibition of IKKβ/NF-κB
	DPP-4 inhibitors	Preserved incretin signalling; modest anti-inflammatory effects involving TLR, IKKβ/NF-κB and JNK pathways
Adiponectin restoration	PPARγ agonists/TZDs	Strongest pharmacological induction of adiponectin through PPARγ activation
	GLP-1 receptor agonists	↑ Adiponectin accompanying improved adipose tissue function and weight loss
	Tirzepatide	↑ Adiponectin with improved visceral adipose tissue phenotype
	SGLT2 inhibitors	Modest ↑ adiponectin
	Metformin	Modest ↑ adiponectin, partly independent of glycaemic control
	Omega-3 fatty acids	Modest improvement in adipokine balance
Leptin resistance reversal	GLP-1 receptor agonists	↓ Visceral adiposity and leptin burden, with improvement in leptin signalling
	Tirzepatide	Substantial reduction in visceral fat and circulating leptin
	SGLT2 inhibitors	↓ Leptin accompanying reduction in adipose tissue mass
	Metformin	↓ Leptin partly related to improved adipose tissue metabolic function
	PPARγ agonists/TZDs	Redistribution from hypertrophic visceral to subcutaneous adipose tissue may improve leptin signalling despite treatment-associated weight gain
M1 → M2 macrophage reprogramming	GLP-1 receptor agonists	↓ Pro-inflammatory macrophage activation and crown-like structures
	Tirzepatide	Combined GLP-1R/GIPR signalling with reduced adipose tissue inflammatory infiltration
	PPARγ agonists/TZDs	PPARγ-dependent anti-inflammatory macrophage reprogramming
	Omega-3 fatty acids	GPR120 signalling and specialised pro-resolving mediators promoting resolution
	Metformin	AMPK-dependent attenuation of M1 inflammatory activation
	SGLT2 inhibitors	↓ M1/M2 macrophage ratio in adipose tissue, ↓ crown-like structures via β-HB-mediated attenuation of IL-1β and NLRP3 signalling
	Statins	↓ Macrophage inflammatory activation
Mitochondrial dysfunction recovery	Metformin	AMPK activation, improved mitochondrial stress adaptation and autophagy
	GLP-1 receptor agonists	↑ AMPK/autophagy and mitophagy, ↓ mitochondrial ROS
	Tirzepatide	Improved mitochondrial substrate utilisation and reduced lipotoxic stress
	SGLT2 inhibitors	Metabolic substrate shift and improved mitochondrial energetic efficiency
	PPARγ agonists/TZDs	Improved adipocyte differentiation and mitochondrial metabolic capacity
	Omega-3 fatty acids	Improved mitochondrial lipid handling and resolution of oxidative stress
Adipose tissue fibrosis	PPARγ agonists/TZDs	Promotion of adipocyte differentiation and attenuation of profibrotic signalling
	GLP-1 receptor agonists	Reduction of adipose tissue inflammation and fibrosis accompanying weight loss
	Tirzepatide	Improved adipose tissue remodelling associated with marked visceral fat reduction
	SGLT2 inhibitors	Reduced adipose tissue inflammatory and hypoxic stress
	Statins	Attenuation of vascular and inflammatory signalling contributing to extracellular matrix remodelling
Adipose tissue expandability	PPARγ agonists/TZDs	Direct pharmacological enhancement of adipogenesis and hyperplastic expansion, increasing capacity for safe lipid storage
	Tirzepatide	GIPR-mediated effects on adipocyte metabolism and adipogenesis, combined with substantial visceral fat reduction
	GLP-1 receptor agonists	Indirect restoration of adipose tissue capacity through sustained reduction of hypertrophic burden
	Metformin	Reduced substrate overload and improved adipocyte metabolic function
	SGLT2 inhibitors	Reduced lipid and energy surplus, indirectly lowering adipose tissue expansion pressure

## Data Availability

No new data were created or analysed in this study. Data sharing is not applicable to this article.

## Abbreviations

The following abbreviations are used in this manuscript: AMPK, AMP-activated protein kinase; β-HB, β-hydroxybutyrate; BMI, body mass index; cAMP, cyclic adenosine monophosphate; CLS, crown-like structures; CR, caloric restriction; CRP, C-reactive protein; CRP-hs, high-sensitivity C-reactive protein; DHA, docosahexaenoic acid; DPP-4, dipeptidyl peptidase-4; DPP-4i, dipeptidyl peptidase-4 inhibitor; ECM, extracellular matrix; EPA, eicosapentaenoic acid; FFA, free fatty acid; GIP, glucose-dependent insulinotropic polypeptide; GIPR, glucose-dependent insulinotropic polypeptide receptor; GLP-1, glucagon-like peptide-1; GLP-1R, glucagon-like peptide-1 receptor; GLP-1RA, glucagon-like peptide-1 receptor agonist; GPR43, G protein-coupled receptor 43; GPR120, G protein-coupled receptor 120; HCAR2, hydroxycarboxylic acid receptor 2; HFpEF, heart failure with preserved ejection fraction; HIF-1α, hypoxia-inducible factor 1α; HOMA-B, homeostatic model assessment of β-cell function; hs-CRP, high-sensitivity C-reactive protein; IKKβ, inhibitor of nuclear factor kappa B kinase subunit beta; IL, interleukin; IMTG, intramyocellular triglyceride; LDL-C, low-density lipoprotein cholesterol; MASH, metabolic dysfunction-associated steatohepatitis; MedDiet, Mediterranean diet; MRI, magnetic resonance imaging; mTORC1, mechanistic target of rapamycin complex 1; NF-κB, nuclear factor kappa B; NLRP3, NOD-, LRR- and pyrin domain-containing protein 3; PGC-1α, peroxisome proliferator-activated receptor gamma coactivator 1-alpha; PKA, protein kinase A; PPARγ, peroxisome proliferator-activated receptor gamma; PVAT, perivascular adipose tissue; PYY, peptide YY; RCT, randomised controlled trial; ROS, reactive oxygen species; RYGB, Roux-en-Y gastric bypass; SCFA, short-chain fatty acid; SGLT2, sodium-glucose cotransporter 2; SGLT2i, sodium-glucose cotransporter 2 inhibitor; SIRT1, sirtuin 1; T2DM, type 2 diabetes mellitus; TGF-β, transforming growth factor beta; TLR4, Toll-like receptor 4; TNF-α, tumour necrosis factor alpha; TXNIP, thioredoxin-interacting protein; TZD, thiazolidinedione; VAT, visceral adipose tissue; VEGF, vascular endothelial growth factor.
